# Supplementary material for: Structural analysis of cancer-relevant TCR-CD3 and peptide-MHC complexes by cryoEM
Source: Nat Commun. 2023 Apr 26;14:2401. doi: 10.1038/s41467-023-37532-7 (PMC10132440; doi:10.1038/s41467-023-37532-7)
Supplement: Supplementary file 1 — Supplementary Information [file 41467_2023_37532_MOESM1_ESM.pdf]

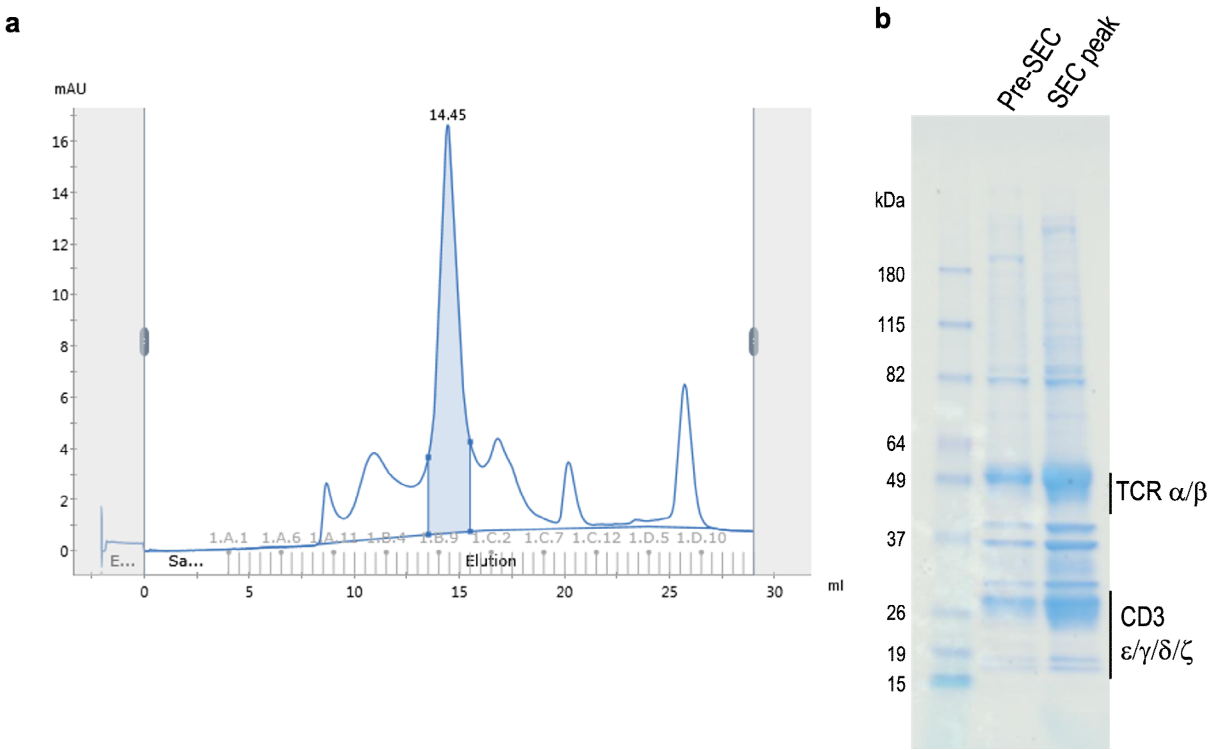

Supplementary Figure 1. Biochemistry of detergent-solubilized TCR-CD3 sample. a, Representative SEC chromatogram (Superose 6 Increase 10/300) of PN45545 TCR-CD3 used for cryoEM. Blue shaded region indicates fractions used for cryoEM sample. Y-axis units in the chromatogram are milli-absorbance units (mAU). **b**, Reducing SDS PAGE gel of PN45545 TCR-CD3. Samples taken before and after the final SEC step were run. Presumed positions of TCR and CD3 subunits are indicated. TCR-CD3 preparations for cryoEM were repeated at least three times with similar results.

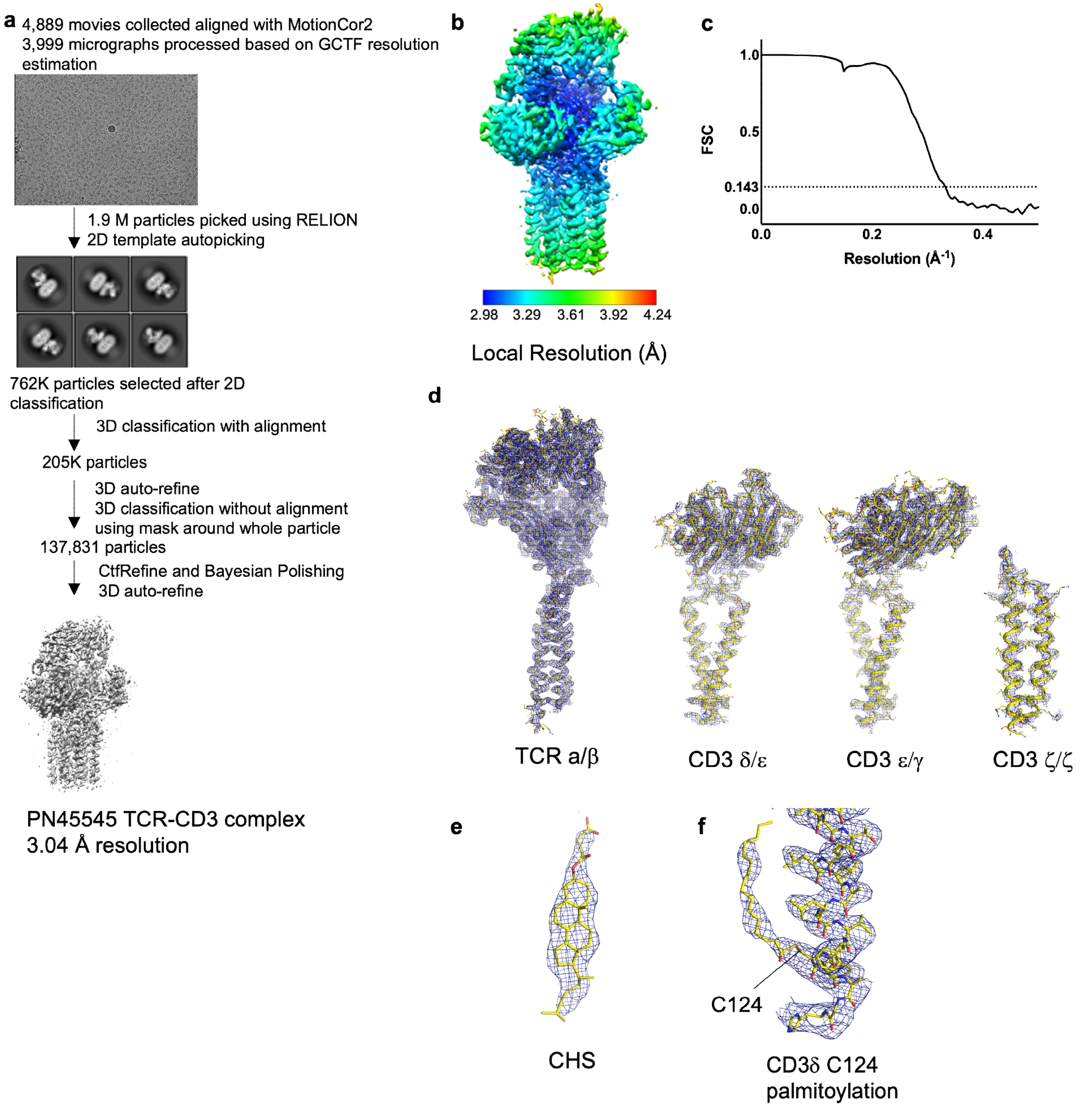

Supplementary Figure 2. CryoEM data processing and reconstruction of PN45545 TCR-CD3. a, Data processing flow chart. **b**, cryoEM map filtered and colored according to local resolution output by RELIOn. **c,** FSC curve output by RELION postprocessing. **d,** Superimposed models and maps of TCR and CD3 subunit dimers. **e,** Fit of a putative cholesteryl hemisuccinate molecule to its density. **f**, CryoEM density protruding out of CD3δ C124 is suggestive of palmitoylation. A fit of the fatty acid to the cryoEM density is shown here for illustration but was not included the deposited model.

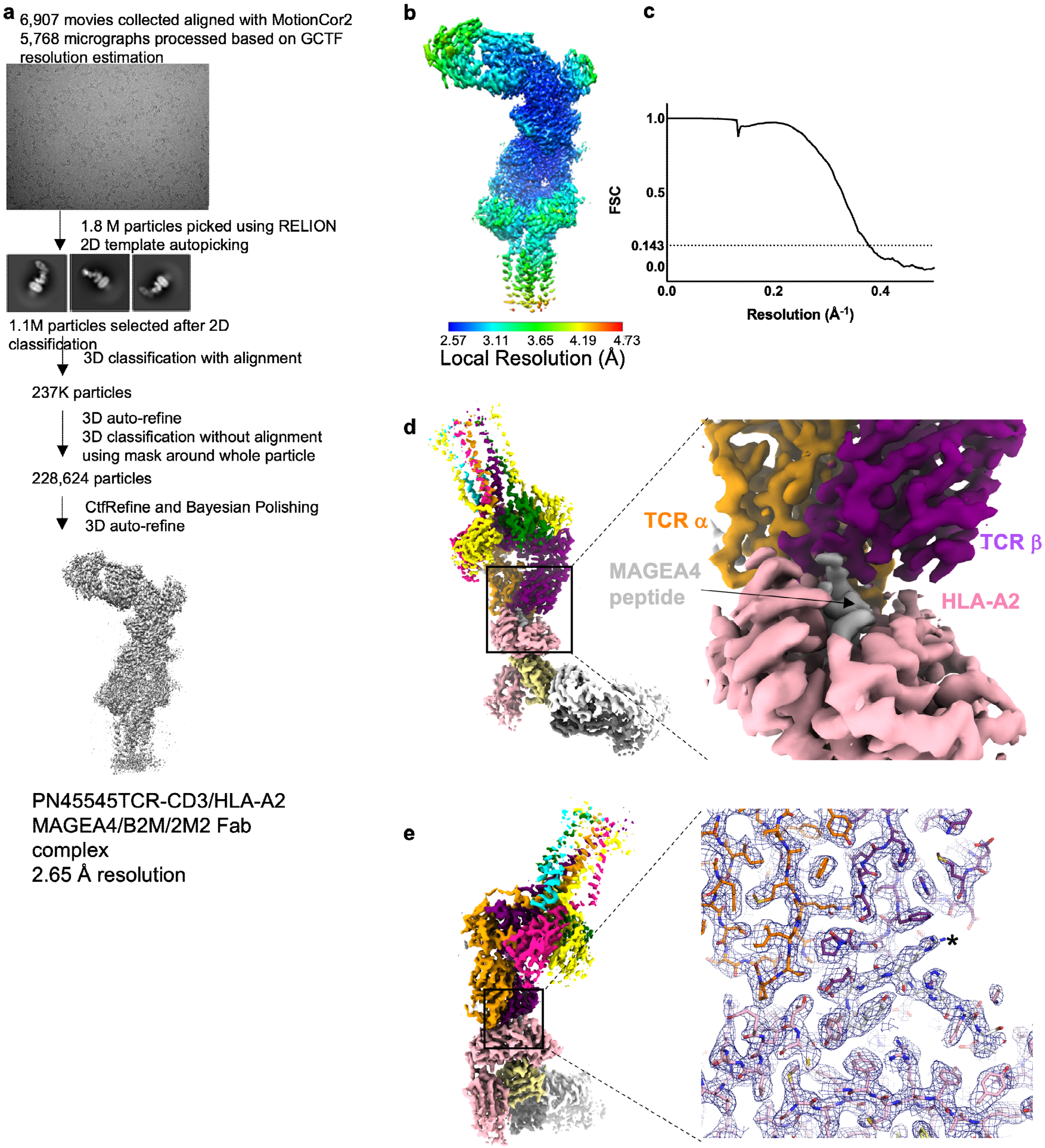

Supplementary Figure 3. CryoEM data processing and reconstruction of PN45545 TCR-CD3 MAGEA4 pMHC complex. a, Data processing flow chart. **b**, cryoEM map filtered and colored according to local resolution output by RELION. **c,** FSC curve output by RELION postprocessing. **d,** tilted side view of PN45545 MAGEA4 TCR-CD3 pMHC complex cryoEM map (left panel), with TCR-CD3 positioned on top and pMHC on bottom. Subunits are colored as in Fig. 2. Right panel shows expanded view of boxed region in left panel**. e,** Left panel shows cryoEM map of PN45545 TCR-CD3 MAGEA4 pMHC complex, colored as in **d** and rotated. Right panel shows sliced view of superimposed model (shown in stick representation and colored by subunit) and map (blue mesh) at the TCR-pMHC interface (boxed region in left panel). Asterisk shows position of MAGEA4 peptide residue R6.

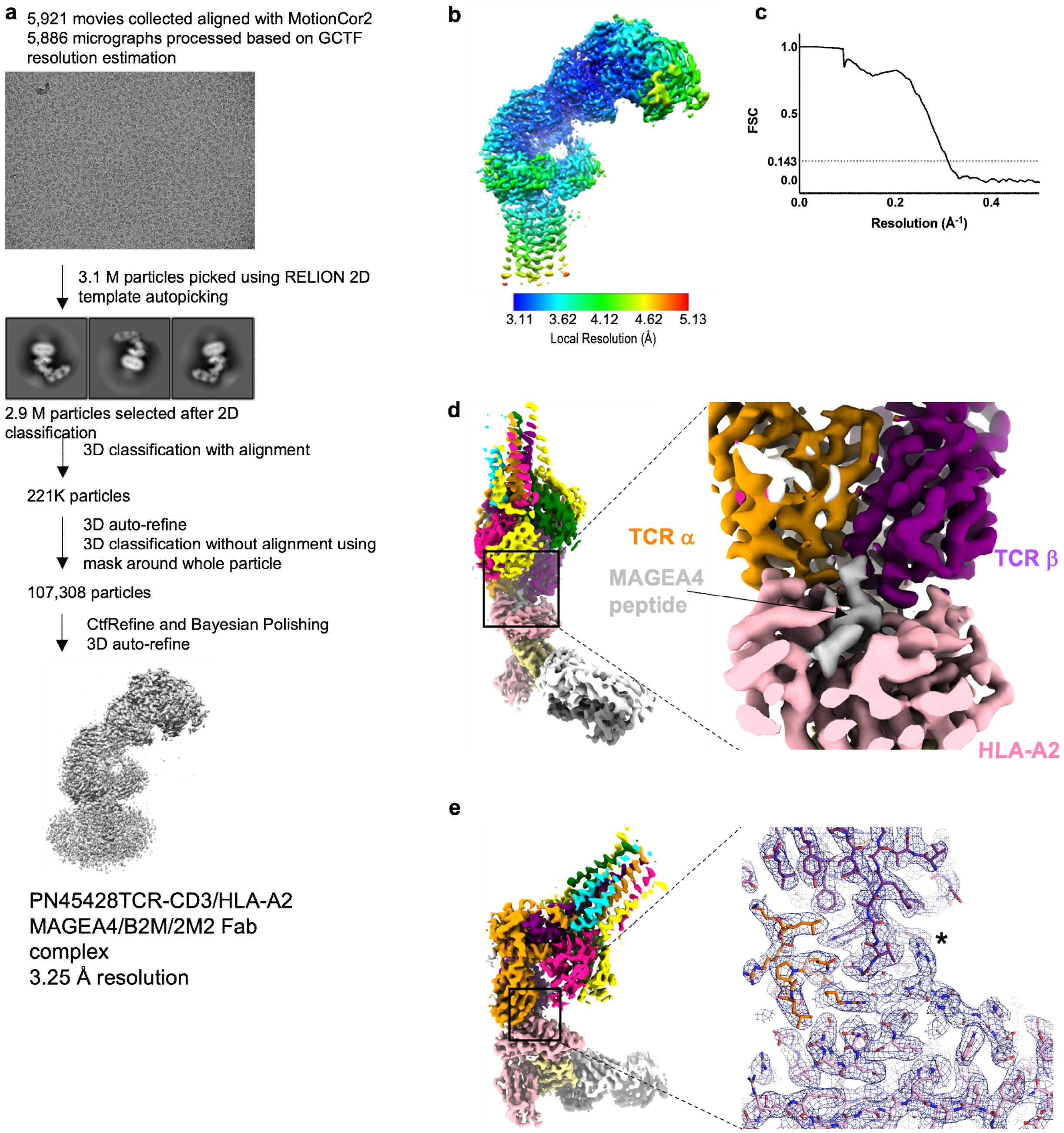

Supplementary Figure 4. CryoEM data processing and reconstruction of PN45428 TCR-CD3 MAGEA4 pMHC complex. a, Data processing flow chart. **b**, cryoEM map filtered and colored according to local resolution output by RELIOn. **c,** FSC curve output by RELION postprocessing. **d,** tilted side view of PN45428 MAGEA4 TCR-CD3 pMHC complex cryoEM map (left panel), with TCR-CD3 positioned on top and pMHC on bottom. Subunits are colored as in Fig. 2. Right panel shows expanded view of boxed region in left panel**. e,** Left panel shows cryoEM map of PN45428 TCR-CD3 MAGEA4 pMHC complex, colored as in **d** and rotated. Right panel shows sliced view of superimposed model (shown in stick representation and colored by subunit) and map (blue mesh) at the TCR-pMHC interface (boxed region in left panel). Asterisk shows position of MAGEA4 peptide residue R6.

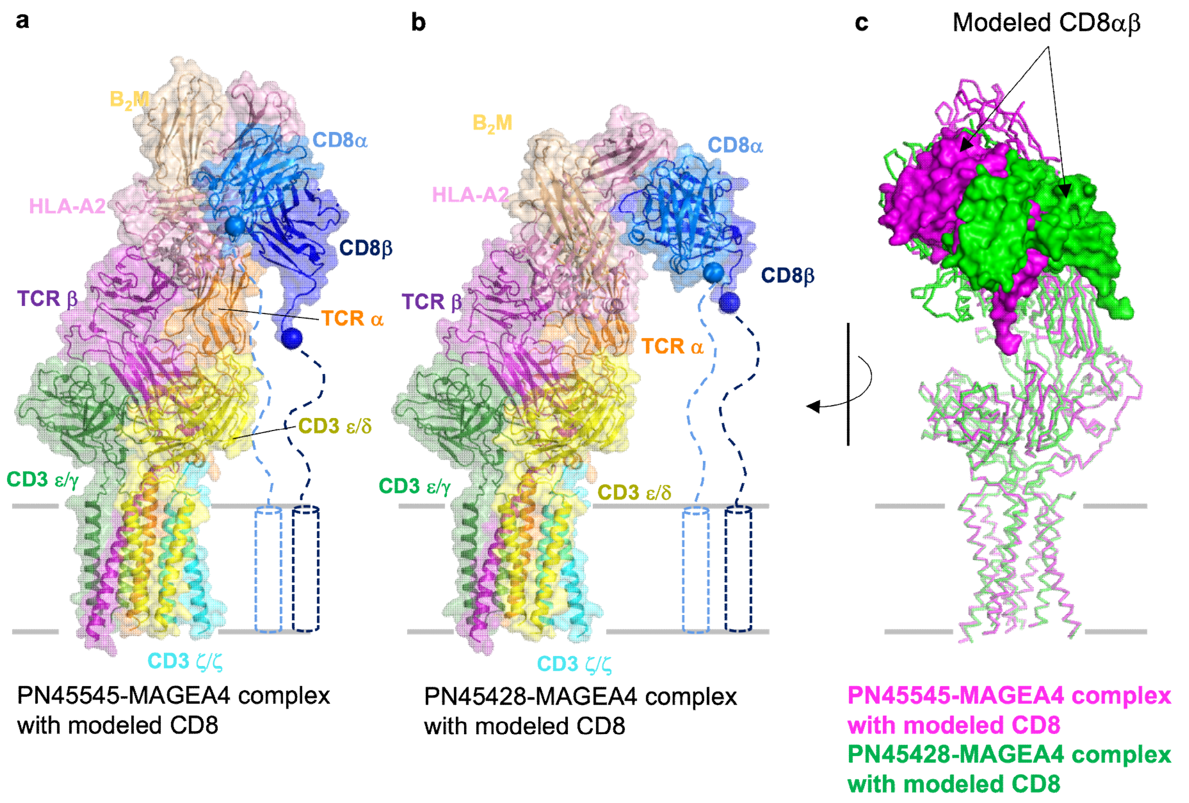

Supplementary Figure 5. Modeling CD8 co-receptor binding onto MAGEA4 TCR complexes. a,b, Cartoon/transparent surface models of PN45545 (a) and PN45428 (b) MAGEA4 TCR complexes with CD8αβ modeled into its MHC binding site. The models were generated by aligning a crystal structure of murine CD8αβ/pMHC complex (PDB 3DMM) with the MAGEA4 pMHC in pymol. Solid horizontal gray lines indicate approximate membrane boundaries. The C-termini of the published CD8αβ crystal structures are depicted as spheres. Flexible stalks connecting the CD8 Ig domains to their transmembrane domains are depicted schematically as dotted lines and cylinders. Note in both models, CD8 is oriented such that their Ig domain C-termini are proximal to the membrane. **c,** Structural comparison of modeled CD8 positions in PN45545 (magenta) and PN45428 (green) TCR/MAGEA4 complexes. The models are rotated such that the CD8 molecules face the viewer. TCR-CD3/MAGEA4 complexes are shown as ribbons and modeled CD8ab molecules are shown as surface.

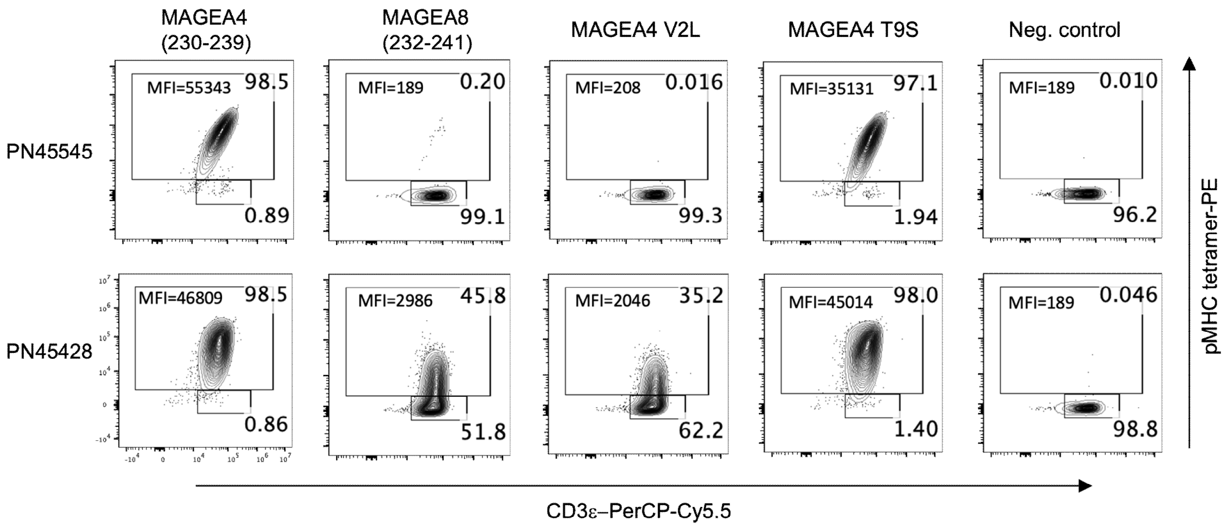

Supplementary Figure 6. Flow cytometry analysis of singly substituted MAGEA4 and MAGEA8 pMHC tetramers binding to Jurkat expressing PN45545 (top row) or PN45428 (bottom row) TCRs. Shown are live, single cells. At least 50,000 live, single cells were acquired for each sample.

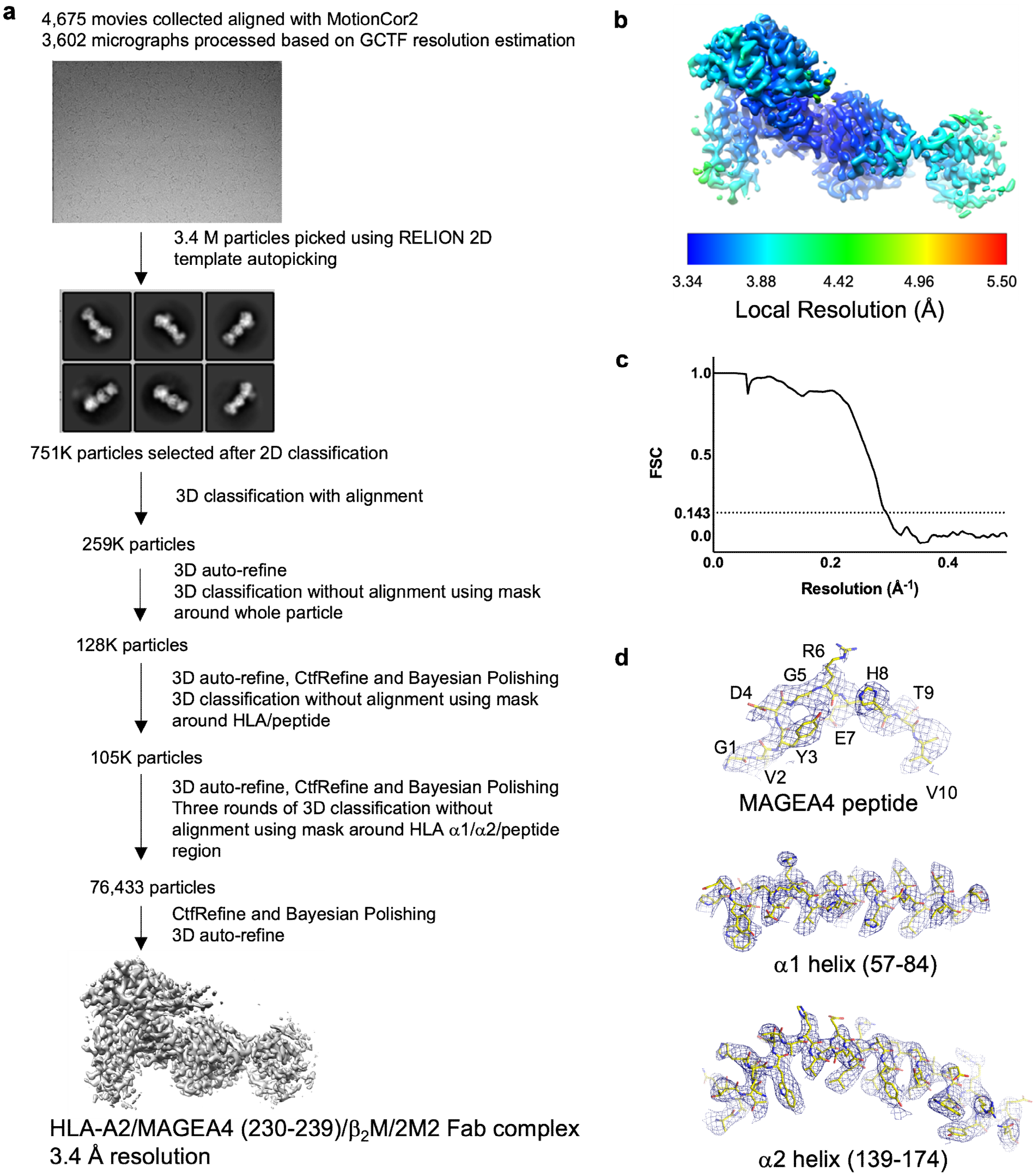

Supplementary Figure 7. CryoEM data processing and reconstruction MAGEA4 pMHC 2M2 Fab complex. a, Data processing flow chart. **b**, cryoEM map filtered and colored according to local resolution output by RELION. **c,** FSC curve output by RELION postprocessing. **d,** Superimposed model (shown in stick representation and colored by subunit) and map (blue mesh) of the peptide, α1 helix, and α2 helix.

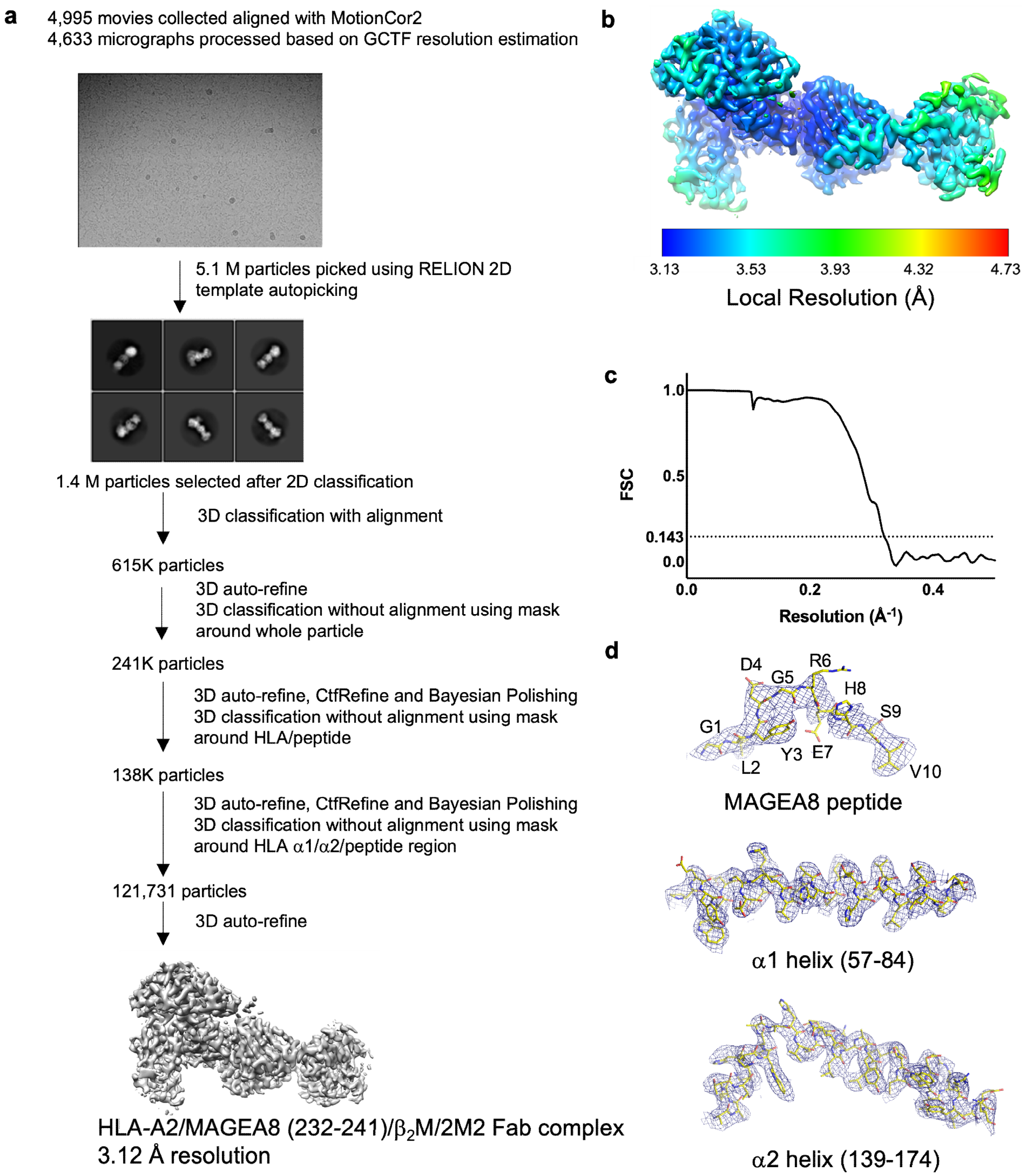

Supplementary Figure 8. CryoEM data processing and reconstruction MAGEA8 pMHC 2M2 Fab complex. a, Data processing flow chart. **b**, cryoEM map filtered and colored according to local resolution output by RELION. **c,** FSC curve output by RELION postprocessing. **d,** Superimposed model (shown in stick representation and colored by subunit) and map (blue mesh) of the peptide, α1 helix, and α2 helix.

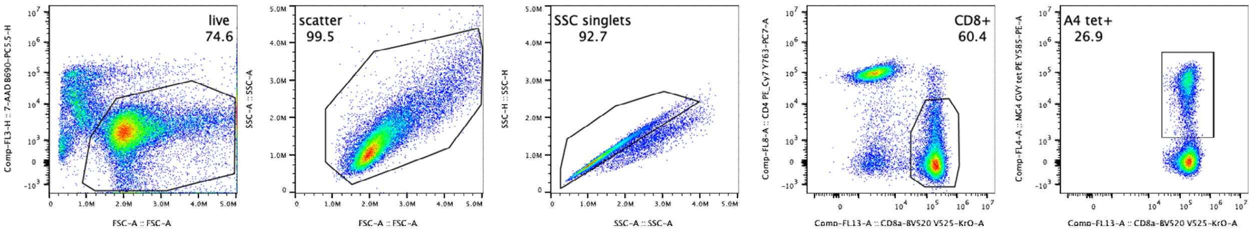

Supplementary Figure 9. Gating strategy for flow experiments shown in Fig. 4a.

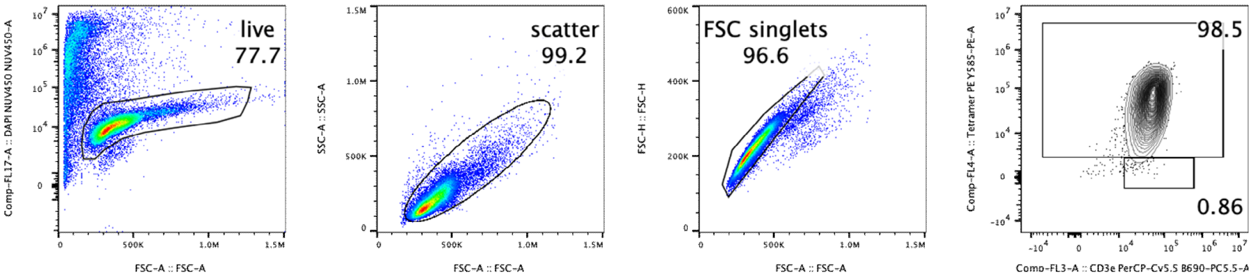

Supplementary Figure 10. Gating strategy for flow experiments shown in Supplementary Fig. 6.

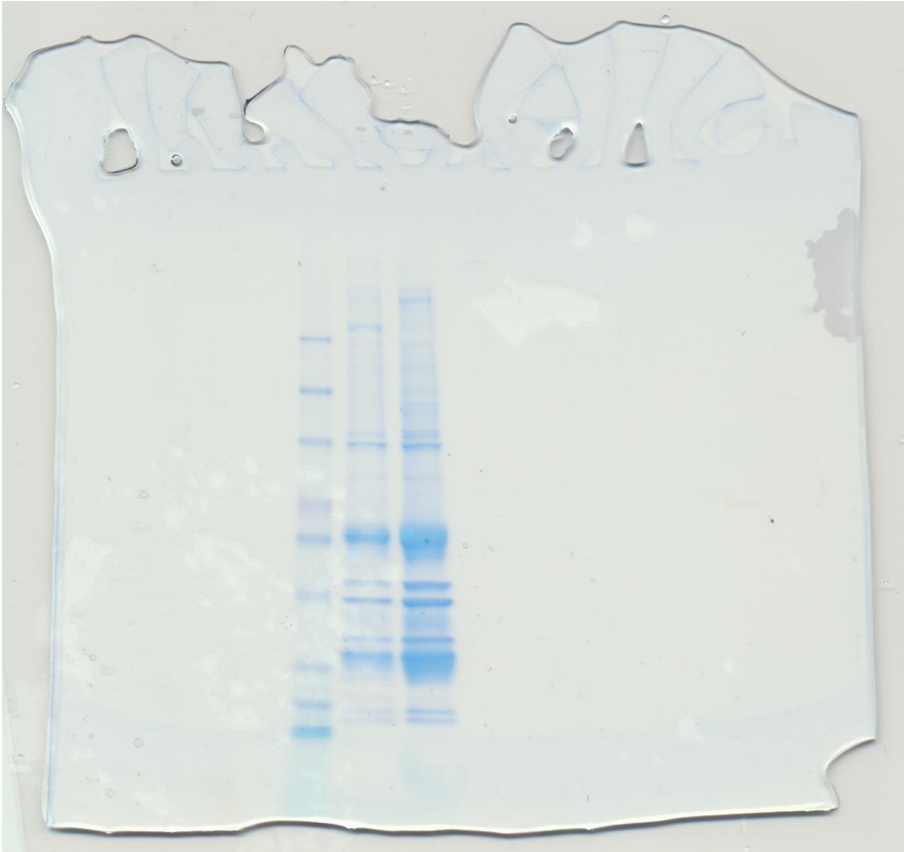

Uncropped gel from Supplementary Figure 1b.
