# Supplementary material for: Structural analysis of cancer-relevant TCR-CD3 and peptide-MHC complexes by cryoEM
Source: Nat Commun. 2023 Apr 26;14:2401. doi: 10.1038/s41467-023-37532-7 (PMC10132440; doi:10.1038/s41467-023-37532-7)
Supplement: Supplementary file 3 — Reporting Summary [file 41467_2023_37532_MOESM3_ESM.pdf]

nature portfolio

Corresponding author(s): Matthew Franklin, Kei Saotome

Last updated by author(s): Mar 8, 2023

Reporting Summary

Nature Portfolio wishes to improve the reproducibility of the work that we publish. This form provides structure for consistency and transparency in reporting. For further information on Nature Portfolio policies, see our Editorial Policies and the Editorial Policy Checklist.

Statistics

For all statistical analyses, confirm that the following items are present in the figure legend, table legend, main text, or Methods section.

n/a	Confirmed	
[ ]	[x]	The exact sample size (n) for each experimental group/condition, given as a discrete number and unit of measurement
[ ]	[x]	A statement on whether measurements were taken from distinct samples or whether the same sample was measured repeatedly
[x]	[ ]	The statistical test(s) used AND whether they are one- or two-sided<br>*Only common tests should be described solely by name; describe more complex techniques in the Methods section.*
[x]	[ ]	A description of all covariates tested
[x]	[ ]	A description of any assumptions or corrections, such as tests of normality and adjustment for multiple comparisons
[ ]	[x]	A full description of the statistical parameters including central tendency (e.g. means) or other basic estimates (e.g. regression coefficient) AND variation (e.g. standard deviation) or associated estimates of uncertainty (e.g. confidence intervals)
[x]	[ ]	For null hypothesis testing, the test statistic (e.g. F, t, r) with confidence intervals, effect sizes, degrees of freedom and P value noted<br>*Give P values as exact values whenever suitable.*
[x]	[ ]	For Bayesian analysis, information on the choice of priors and Markov chain Monte Carlo settings
[x]	[ ]	For hierarchical and complex designs, identification of the appropriate level for tests and full reporting of outcomes
[x]	[ ]	Estimates of effect sizes (e.g. Cohen's d, Pearson's r), indicating how they were calculated

Our web collection on statistics for biologists contains articles on many of the points above.

Software and code

Policy information about availability of computer code

Data collection	As described in the methods: CryoEM data collected using EPU 2.12, flow data collected using CytExpert 2.5, SPR data collected using T200 control software 3.2.1 (Cytiva). No custom code used.
Data analysis	As described in the methods: CryoEM data analyzed using Relion 3, cryoSPARC v2, Coot 0.8.9, Phenix 1.19. Flow data analyzed using FlowJo 10.5.2, SPR data analyzed using Scrubber v2.0. No custom code used.

For manuscripts utilizing custom algorithms or software that are central to the research but not yet described in published literature, software must be made available to editors and reviewers. We strongly encourage code deposition in a community repository (e.g. GitHub). See the Nature Portfolio guidelines for submitting code & software for further information.

Data

Policy information about availability of data

All manuscripts must include a data availability statement. This statement should provide the following information, where applicable:

- Accession codes, unique identifiers, or web links for publicly available datasets
- A description of any restrictions on data availability
- For clinical datasets or third party data, please ensure that the statement adheres to our policy

Regeneron materials described in this manuscript may be made available to qualified, academic, noncommercial researchers through a materials transfer agreement upon request at https://regeneron.envisionpharma.com/vt_regeneron/. For questions about how Regeneron shares materials, use the email address preclinical.collaborations@regeneron.com. Structural coordinates have been deposited to the Protein Data Bank (PDB) and cryoEM maps have been deposited to

the Electron Microscopy Data Bank (EMDB) with accession numbers 8ES7 [http://doi.org/10.2210/pdb8ES7/pdb] and EMD-28570 [https://www.ebi.ac.uk/pdbe/entry/emdb/EMD-28570] (PN45545 TCR-CD3), 8ES8 [http://doi.org/10.2210/pdb8ES8/pdb] and EMD-28571 [https://www.ebi.ac.uk/pdbe/entry/emdb/EMD-28571] (PN45545 TCR-CD3 in complex with MAGEA4 pMHC), 8ES9 [http://doi.org/10.2210/pdb8ES9/pdb] and EMD-28572 [https://www.ebi.ac.uk/pdbe/entry/emdb/EMD-28572] (PN45428 TCR-CD3 in complex with MAGEA4 pMHC), 8ESA [http://doi.org/10.2210/pdb8ESA/pdb] and EMD-28573 [https://www.ebi.ac.uk/pdbe/entry/emdb/EMD-28573] (MAGEA4 pMHC in complex with 2M2 Fab), 8ESB [http://doi.org/10.2210/pdb8ESB/pdb] and EMD-28574 [https://www.ebi.ac.uk/pdbe/entry/emdb/EMD-28574] (MAGEA8 pMHC in complex with 2M2 Fab).

Human research participants

Policy information about studies involving human research participants and Sex and Gender in Research.

Reporting on sex and gender	No human research is reported in this manuscript.
Population characteristics	Not applicable
Recruitment	Not applicable
Ethics oversight	Not applicable

Note that full information on the approval of the study protocol must also be provided in the manuscript.

Field-specific reporting

Please select the one below that is the best fit for your research. If you are not sure, read the appropriate sections before making your selection.

[x] Life sciences [ ] Behavioural & social sciences [ ] Ecological, evolutionary & environmental sciences

For a reference copy of the document with all sections, see nature.com/documents/nr-reporting-summary-flat.pdf

Life sciences study design

All studies must disclose on these points even when the disclosure is negative.

Sample size	Numbers of particles in the cryoEM samples are described in the methods. SPR data used two independent experiments.
Data exclusions	CryoEM data processing, including which particles are included or excluded from the final map, is described in the methods. Flow gates are shown in a supplementary figure as requested. No data were excluded from the SPR analysis.
Replication	CryoEM experiments were not repeated. SPR data was replicated once (two independent experiments) and reported. Flow data was replicated twice for MAGEA4 and MAGEA8 samples, but only one experiment was run for the A4/A8 chimeric constructs due to time and materials constraints.
Randomization	CryoEM particles were randomly assigned to two half-sets, following the "gold standard" FSC protocol. Randomization is not relevant to the flow cytometry and SPR experiments, as all data were used in a single analysis procedure.
Blinding	Blinding is not relevant to the cryoEM, flow cytometry, and SPR techniques used in this study; none of the information collected and processed is subject to experimenter bias in a way that blinding would address.

Reporting for specific materials, systems and methods

We require information from authors about some types of materials, experimental systems and methods used in many studies. Here, indicate whether each material, system or method listed is relevant to your study. If you are not sure if a list item applies to your research, read the appropriate section before selecting a response.

Materials & experimental systems

n/a	Involved in the study	
[ ]	[x]	Antibodies
[ ]	[x]	Eukaryotic cell lines
[x]	[ ]	Palaeontology and archaeology
[x]	[ ]	Animals and other organisms
[x]	[ ]	Clinical data
[x]	[ ]	Dual use research of concern

Methods

n/a	Involved in the study	
[x]	[ ]	ChIP-seq
[ ]	[x]	Flow cytometry
[x]	[ ]	MRI-based neuroimaging

Antibodies

Antibodies used	CryoEM experiments used 2M2 mouse IgG1 antibody (BioLegend cat#316302). Flow experiments used staining antibodies against human CD3ε (RRID:AB_2744387, Brilliant Ultraviolet 395, cl. UCHT1, BD 563546, final dilution 1:50), human CD8α (RRID:AB_2561942, Brilliant Violet 510, cl. RPA-T8, BioLegend 301048, final dilution 1:200), and human CD4 (RRID:AB_314080, PE-Cyanine7, cl. RPA-T4, BioLegend 300512, final dilution 1:200).
Validation	Antibody validations provided by the relevant manufacturers. BioLegend states "Purity testing and molecular mass determined by SDS-PAGE. Flow cytometry specificity testing of 1-3 target cell types with either single- or multi-color analysis (including positive and negative cell types)." BD states "Flow cytometry specificity is confirmed using multiple methodologies that may include a combination of flow cytometry, immunofluorescence, immunohistochemistry or western blot to test staining on a combination of primary cells, cell lines or transfectant models." No additional validation was done by the authors of this manuscript.

Eukaryotic cell lines

Policy information about cell lines and Sex and Gender in Research

Cell line source(s)	HEK293F and Sf9 cells for cryoEM protein production came from Thermo Fisher. Jurkat E6 cells came from ATCC. Primary human T cells (healthy female donor) came from StemExpress as described in the methods. CHO-K1 derived cells are from Regeneron stocks.
Authentication	Jurkat cells were confirmed by STR profiling. HEK293F and Sf9 cells were not authenticated. CHO-K1 derivative cells were confirmed by whole genome sequencing.
Mycoplasma contamination	Jurkat cells and CHO-K1 derivative cells were confirmed to be mycoplasma free. HEK293F and Sf9 cells were not tested for mycoplasma.
Commonly misidentified lines (See ICLAC register)	No commonly misidentified cell lines were used in this study.

Flow Cytometry

Plots

Confirm that:

- [x] The axis labels state the marker and fluorochrome used (e.g. CD4-FITC).
- [x] The axis scales are clearly visible. Include numbers along axes only for bottom left plot of group (a 'group' is an analysis of identical markers).
- [x] All plots are contour plots with outliers or pseudocolor plots.
- [x] A numerical value for number of cells or percentage (with statistics) is provided.

Methodology

Sample preparation	TCRs were expressed in primary human T cells by targeting AAV-encoded TCR constructs to the TRAC locus. Three days after activation, beads were removed, and cells were nucleofected with Crispr RNP consisting of Cas9 protein (Life Tech A36499) complexed with a mixture of modified synthetic guide RNAs (sgRNAs, IDT) targeting the TRAC (GCUGGUACACGGCAGGGUCA) and TRBC1/2 (UGGGAAGGAGGUGCACAGUG) genes in their first exons. 5e6 T-cells were suspended in 100 ml nucleofection buffer (Lonza VPA-1002) containing 30 mg Cas9 complexed with 150 pmol of each sgRNA, and electroporated with the T-020 program on the Lonza Nucleofector IIb. Cells were transferred immediately into media containing adeno-associated virus (AAV, 4e4 viral genomes/cell) vectors encoding homology directed repair templates for TRAC insertion. Every 2-3 days, cells were diluted to 0.5-1e6 cell/ml in media with fresh cytokines. TCR expression and antigen binding was evaluated by flow analysis with pHLA tetramers.
Instrument	BC CytoFLEX LX
Software	Flow cytometry data were acquired using CytExpert 2.5 and analyzed using FlowJO 10.5.2. No custom code used.
Cell population abundance	not applicable to the flow analysis reported
Gating strategy	Gating strategies are shown in supplementary figures.

- [x] Tick this box to confirm that a figure exemplifying the gating strategy is provided in the Supplementary Information.
